# Super-resolution deep neural network (SRDNN) based multi-image steganography for highly secured lossless image transmission

**DOI:** 10.1038/s41598-024-54839-7

**Published:** 2024-03-13

**Authors:** S. Priya, S. P. Abirami, B. Arunkumar, B. Mishachandar

**Affiliations:** 1 Present Address: Department of Computer Science and Engineering, Coimbatore Institute ofTechnology, Coimbatore, India; 2 School of Computer Science and Engineering, VIT-AP, Amaravathi, India

**Keywords:** Steganography, Cryptography, Deep neural networks, Encryption, Decryption, Medical research, Engineering, Medical research, Engineering

## Abstract

Information exchange and communication through the Internet are one of the most crucial aspects of today’s information technology world. The security of information transmitted online has grown to be a critical concern, particularly in the transfer of medical data. To overcome this, the data must be delivered securely without being altered or lost. This can be possibly done by combining the principles of cryptography and steganography. In the recent past, steganography is used with simpler methods like the least significant bit manipulation technique, in order to encode a lower-resolution image into a higher-resolution image. Here, we attempt to use deep neural networks to combine many two-dimensional colour images of the same resolution into a single cover image with the same resolution. In this technique, many secret images are concealed inside a single cover image using deep neural networks. The embedded cover image is then encrypted using a 3D chaotic map for diffusion and elliptic curve cryptography (ECC) for confusion to increase security.Supporting the fact that neural networks experience losses, the proposed system recovers up to 93% of the hidden image concealed in the original image. As the secret image features are identified and combined along with the cover image, the time complexity involved in the security process is minimized by 78% compared to securing the original data.

## Introduction

Information security has become an inseparable part of data communication as the volume of data shared over the internet has increased. It has resulted in an enormous growth in the field of information concealment. There are several methods for concealing information, two of which are cryptography and steganography. Steganography is the process of concealing secret information within non-secret information. There are different types of steganographic techniques available such as text, audio, image, video and network steganography. The goal here is to perform steganography on images. Image steganography entails concealing a secret, full-size, colour image within another, non-secret image known as the cover image. As one of the most serious issues in digital communication is the security and integrity of data communicated over the internet network, the cryptographic technique is embedded with steganography. Cryptography is the science of hiding information to keep it secret from unauthorised people. Previously, this technology was limited to the encryption and decryption of messages sent using secret keys, but security is rapidly improving.

Using specific algorithms, the stego-system can conceal the secret information in the cover media. A secret message could be an image, text, audio, video, or anything else that can be represented in bits. Following the embedding of the secret data in the cover image, also known as a stego-object, sending to the receiver begins by selecting the appropriate channel, where the decoder system is used with the same stego-method to obtain original information from the sender. In this case, SRDNN is used in conjunction with steganographic and cryptographic techniques to improve the spatial resolution of an image, with interpolated low-resolution images serving as input. When compared to traditional interpolated methods such as bilinear, nearest neighbour, bicubic, cubic, and lanczos4, it produced promising results. Furthermore, the accuracy of disease classification using AlexNet architecture increased by 20% between low and super-resolution images, while the accuracy increased by 90%. Few other research papers focused on classic work to delve deeper into super-resolution deep neural networks (SRDNN) and demonstrated improved performance in significant research fields such as plant disease diagnosis, cytogenetics, blood cell count, CT scan, vehicle detection, agricultural pest detection, and many.

To increase the security involved in the embedded image for transmission, a 3D chaotic mapping is involved. A 3D chaotic map is a mathematical model that displays chaotic behaviour in three dimensions. Chaotic maps are nonlinear dynamical systems distinguished by their sensitivity to beginning conditions, unpredictability, and complicated behaviour. These characteristics make them useful in a variety of scientific and engineering applications, including cryptography, data encryption, secure communications, picture processing, and random number generation. The proliferation of digital images and the necessity to secure sensitive visual information have prompted the development of improved cryptographic algorithms for image security in recent years. Elliptic curve cryptography (ECC), a branch of public-key cryptography that provides solid security and efficient encryption processes, is one such powerful approach. ECC provides a safe framework for picture encryption and decryption by exploiting the mathematical features of elliptic curves, ensuring confidentiality and integrity during data transport and storage. The primary goal of this research is to propose a hybrid security system for ensuring data security and resolving the capacity problem of data exchanged over the internet, by using an intelligent mix of algorithms to hide a sensitive image inside another.This manuscript covers the cryptography part that is proposed as a super-resolution deep neural network.

### Summary

The remaining paper is sectioned as follows. Section "State of art" covers the recent state of the art broadly discussing the recent contribution to literature and Sect. "Proposed methodology" with the proposed methodology. The experimental results with discussion and the conclusion are covered in Sects. "Experimental results and evaluations" and "Conclusion".

## State of art

The Internet has evolved into a significant source of information transfer, online shopping, reservations, and payments, among other things^1^. Secure information is required with this advancement in online information transfer to avoid interception from unauthorised interceptors. Image steganography has traditionally been used by researchers to conceal information from humans. Some of the algorithms developed in the previous studies performed well. They are gradually improved after the encoding process to limit physical modification properties on the images^2^. As a result, this scenario necessitates a research requirement that focuses on using image steganography while maintaining efficiency by using a convolution-based neural network to fix the image resolution. Integrating steganography methods and neural networks is critical because it works in real-time and addresses the most commonly used neural network techniques in today’s systems^3^. The primary goal of this work is to implement image steganography techniques with higher resolution while maintaining system security. In many application domains, real-time input images with low resolution were provided for experimental study. The use of these images appears to be the primary cause of the relatively poor diagnostic performance, and thus low image quality has been identified as an important factor influencing classification accuracy^4,5^. The goal is to improve the quality of low-resolution images, which is an important step in emerging research domains such as security surveillance, agriculture, medical diagnostics, remote sensing, ultrasound imaging, astronomical observation, and biometric recognition, among others^6^.

Image SR techniques aims to improve the image resolution from single or multiple images.SR based on a single image, known as Single Image Super Resolution (SISR), has recently demonstrated significant performance using learning and reconstructed methods^7^. Authors Dong et al. have employed pre-up sampling SR techniques as well as proposed a Convolutional Neural Network-based Super Resolution model known as the SRDNN model, which demonstrates significant prediction performance on mapping of low resolution and reconstructed high-resolution images. Furthermore, larger filter sizes are used in CNN layers, and the performance of SRDNN is compared to conventional methods. In a similar context relating to the design of a deep neural network to perform multi-image steganography authors Abhishek Das, Yugant Rana, and Mansi Anand in Ref.^8^ have proposed a Deep Neural Networks model to perform Multi-Image Steganography. The methodology of hiding is extended using a single secret image inside a single cover image to hiding multiple secret images inside a single cover image. Multiple preparation networks are used to hide multiple secret images, and multiple revealing networks are used to decode them. The inference observed form the state of art is that deep neural networks were used to conceal and reveal multiple secret images with minimal loss. By using one image as a cover image to conceal several secret images and retrieving them with little loss, is the improvisation planned for the system.

Authors Shumeet Baluja et al. in Ref.^9^ have proposed a model for image steganography. With little distortion to cover images, a N x N x RGB pixel secret image is concealed inside another N x N x RGB pixel cover image. Many network layers, including the Preparation Network, Hiding Network, and Reveal Network, which are used to hide images, are utilised in the model design of the proposed system.

Steganography is a vast field of study with a huge scope for research with relevance to neural networks. Authors Nandhini Subramanian et al. in Ref.^10^ have examined the various Steganography methods available throughout history and discussed their benefits and drawbacks. The paper also discusses the various datasets available, evaluation metrics, and challenges associated with Steganography. This paper is based on the different methods detailed in this paper and decided to work with a recent method (i.e. the use of neural networks in Steganography)^11^. One of the simplest methods of Steganography in the concept of Steganography is the Least Significant Bit. The Least Significant Bit (LSB) method hides the message by inserting the message at the lower or rightmost bits in the cover work file as a medium to hide the message^12^. Vikas Singhal, Yash Kumar Shukla, and Navin Prakash^13^ proposed a method that uses the LSB technique in order to replace the least significant bits in the cover image which contains bits of the hidden message.

Singh A. K. et al. proposed the importance of confusion and diffusion strategies for image encryption. Research describes a new encryption technique that can encrypt both greyscale and colour medical photos^14^. Similarly, Yue Wu et al. have suggested local Shannon entropy measure overcomes several shortcomings of the traditional global Shannon entropy measure, such as unfair randomness comparisons between images of varying sizes, the inability to distinguish between image randomness before and during image shuffling, and potentially erroneous results for synthetic images^15^.

To create pseudo-random keys for encryption, the author proposes an approach that employs a chaotic 3D Lorenz attractor and logistic map. According to their findings, the IWT-based chaos-DNA approach is effective for safeguarding medical pictures in a variety of real-time medical applications. With the advent of the chaotic advantage, the proposed method adopts it to bring maximum security^16^.

Due to the possible security issue of key management and distribution for symmetric image encryption systems, Luo Y. et.al. proposed a novel asymmetric image encryption approach based on elliptic curve ElGamal (EC-ElGamal) cryptography and chaos theory^17^. Specifically, the SHA-512 hash is utilised to construct the initial values of a chaotic system before scrambling the plain image with a crossover permutation in terms of the chaotic index sequence. Furthermore, the created scrambled picture is integrated into the elliptic curve for the encryption by EC-ElGamal, which not only improves security but also aids in the resolution of key management issues. Finally, the cypher image is obtained by playing the diffusion mixed chaos game with the DNA sequence. The literature proves and paves the way for the proposed research to improve security.

The limitations of the state-of-the-art firstly define that the computational complexity along with time complexity will be more in the existing methodology. This is because the input images involved in the process will be of a good quality pixel image and thus involve more computational cycles. Secondly, the images are secured using chaotic key mapping which brings more security than the existing algorithms involved in secure transmission. Though steganography involves embedding secret images for secure transmission, the amount of time and cost involved in securing and transmission is high which degrades the accuracy and performance of the system. Firstly, the main aim of the proposed system is to identify the important features of any image along with the cover and is embedded only with those identified features, the weightage of the important features is highlighted and thus computational complexity is reduced. This brings the importance of involving the neural network in the steganographic technique. Secondly, cryptographic techniques are involved to securely transmit the embedded image over the network without compromising the security threats. Finally, this integrated proposed architecture takes advantage of all the emerging field technologies to attain maximum accuracy in its outcome. Leveraging steganographic techniques can add hidden layers of encryption within the image, while the cryptographic aspect ensures the strength of the encryption key. Incorporating neural networks can bring in additional complexity to make the encryption more resilient against attacks. The combined complexity of steganography, cryptography, and neural networks makes the encrypted image more resistant to various attacks.

The research introduced a novel image-splitting technology based on image blocks. The image blocks were then scrambled with a zigzag pattern, rotation, and random permutation. The scrambled image is then diffused using a chaotic logistic map. Our proposed method’s efficacy in encrypting medical images is tested by utilising security analysis and time complexity. Entropy, histogram differential attacks, correlation coefficient, PSNR, key-space, and sensitivity are all investigated. The obtained results demonstrated a high-performance security level attained by successful encryption of both grey and colour medical photos.

## Proposed methodology

**Figure 1:**
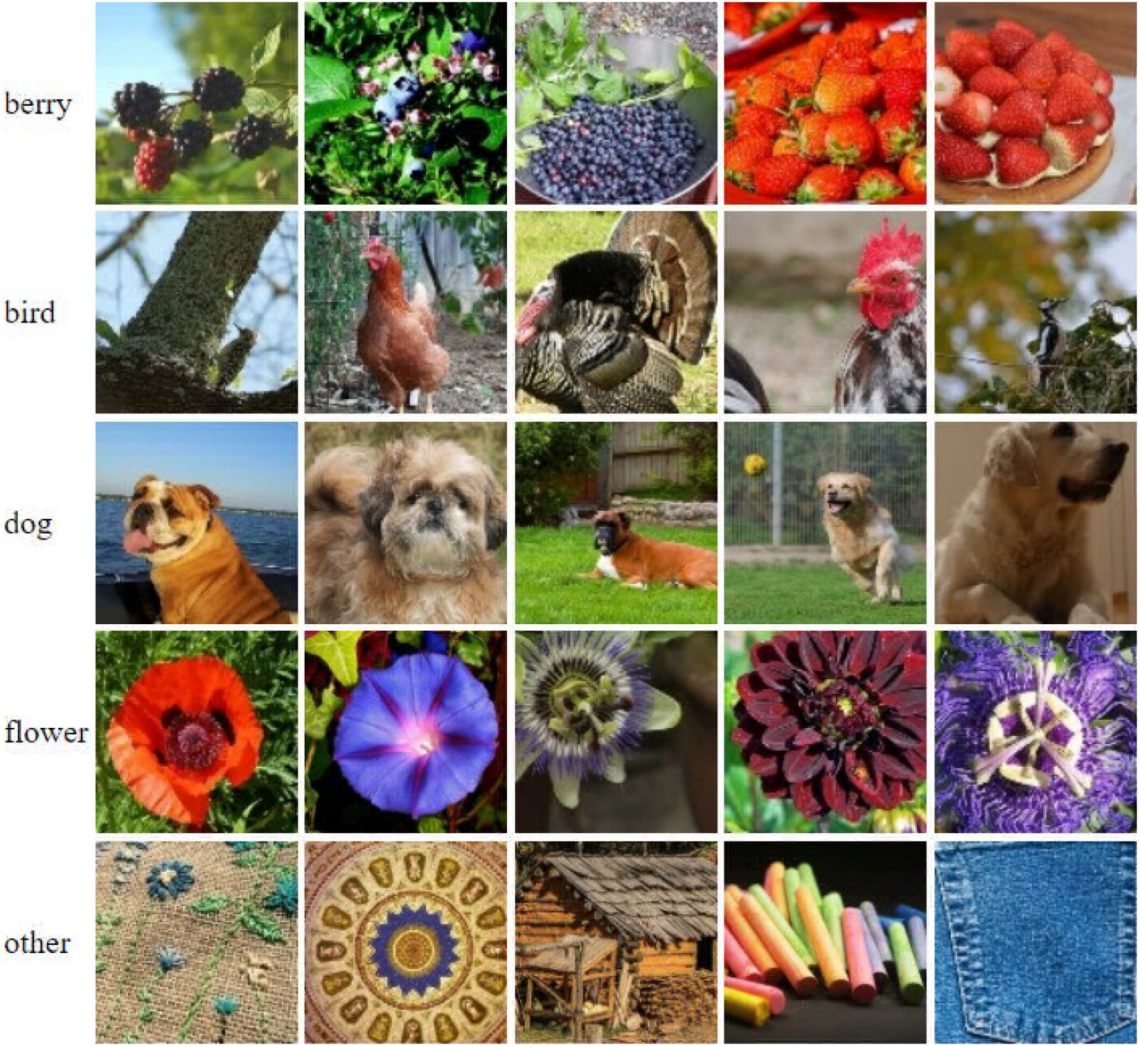
Testing sample of the Linnaeus 5 dataset.

The proposed methodology of SRDNN is tested with the Linnaeus 5 Dataset which is available open source. This dataset is divided into five classes. Flower, Dogs, Birds, Berry, and Others are their names^18^. The images in this dataset are 256 x 256 in size. This dataset includes 1200 training images and 400 test images for each class. The following are some examples of images from this dataset. The model architecture of the proposed SRDNN system is depicted in Fig. 1.

### Super-resolution-based image transformation

**Figure 2:**
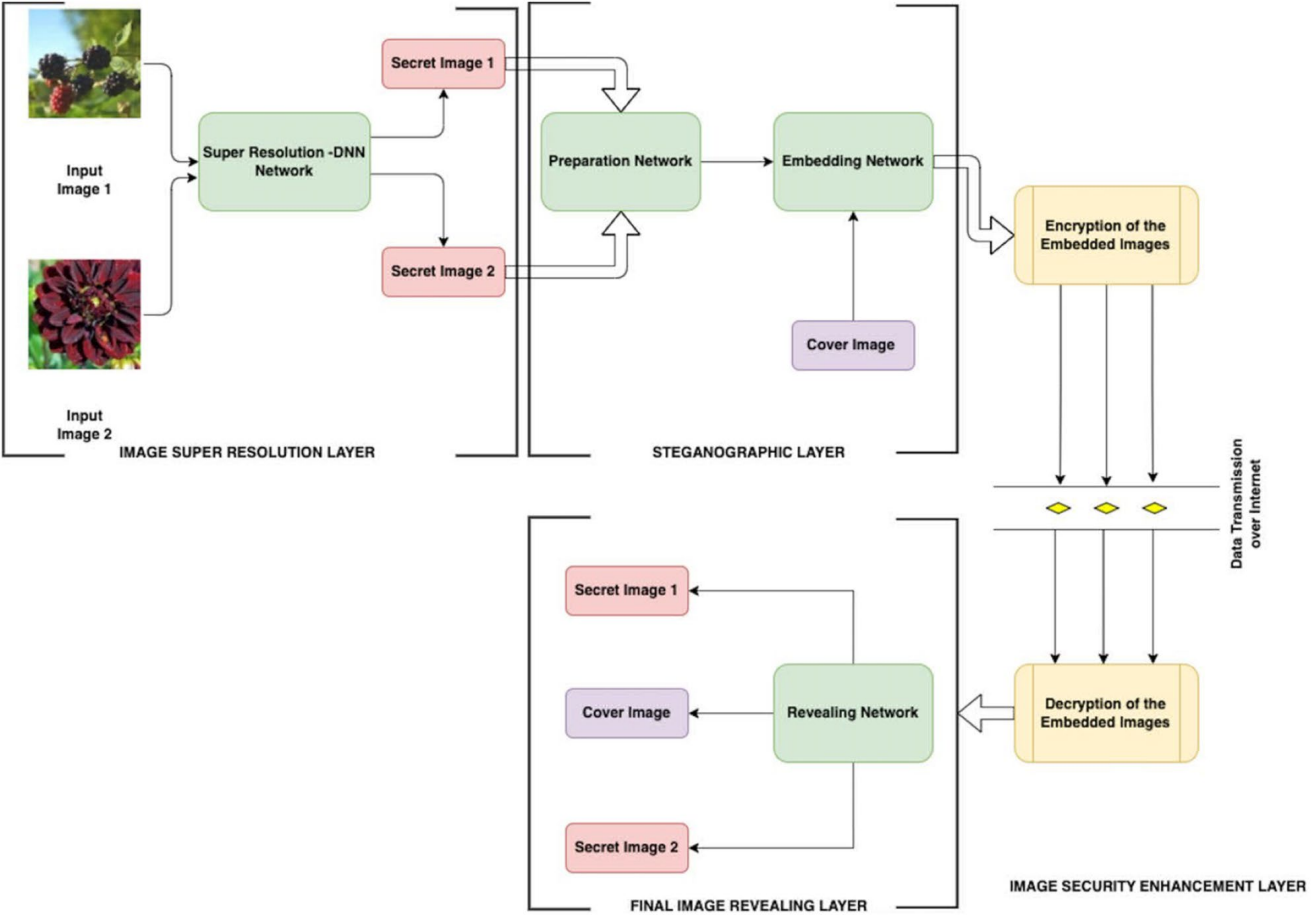
Proposed architecture of super-resolution deep neural network (SRDNN).

The paper aims to propose a super-resolution image SRi from the low-resolution image LRi and learn a function mapping between SRi and LRi.The quality of the image is recovered using a deep neural network (DNN) and a Super-resolution model is proposed for image multi-image steganography. To upscale the Low-Resolution image to the Super-Resolution image size, the SR-DNN employs a three-layer convolution network, with the up-sampling layer applied before the convolutional layers. LRi is the low-resolution version of HRi that includes tensor properties such as width (w), height (h), and RGB colour channel with an input colour image (Ci). In general, up-sampling methods such as bilinear, bicubic, nearest neighbour, cubic, deconvolution, and unspooling. The SR-DNN uses bicubic interpolation for upscaling the image to its desired size for further computation. Up-sampling layers in the proposed work use bilinear and nearest neighbour interpolation methods to achieve better results for embedding into the cover image. Figure 2 shows the proposed architecture of the super-resolution deep neural network.

Before feeding the convolutional block, the output of bilinear interpolated images is concatenated with the nearest neighbour interpolated input. The upscaled low-resolution image as LRi will be used as an input for the neural network’s first layer. Consider the input image LRi, which has the dimensions w, and h, which represent the width and height, respectively. LRi is convoluted with kernels of size f1 x f1, and its depth is n1, implying that the number of filters in this layer equals n1. Using the bilinear interpolation technique, the first layer of the network is scaled down and then scaled up to the input RGB image. Using the 9 x 9 kernel function, this layer extracts n1 dimensional feature maps (n1=128). Similarly, the second layer uses n2 filters and kernel sizes such as f2 x f2 to map n1 to n2 dimensional feature maps. W2 and B2 are the kernel size and bias vector in the second layer, respectively, and L1(ILR) is the feature map from the first layer. The W2 is n1 x f2 x f2 because f2 is set to 3 and bias vector n2 is set to 64. The second layer’s output is depicted as n2-dimensional vectors and thus represents the high-resolution patch that will be used for image reconstruction. The third layer of the network aggregates the preceding output’s high-resolution patches, leading to a final super-resolution image. W3 is the kernel size in filter n3, and the size is given as n2 x f3 x f3 c, where f3=5 and B3 is the c dimensional vector size, with c = 1.

### Multi-image steganography using SRDNN

The primary goal is to carry out multi-image steganography, in which two or more images are concealed under a single cover image. The embedded hidden photos are then extracted with as little loss as possible. The encoded cover image must resemble the original cover image in appearance. Implementing the network with advanced planning, concealing the network as an encoder, and employing the exposed network as a decoder could have positive results. To extend this for numerous photos, the preparation network is used to send several secret images, and the resulting data is then concatenated with the cover image. It then travels via the Hiding network. The idea of employing multiple decoders-one decoder for each secret image-was then what we intended to use to extract all the secret images from the embedded cover image. With the use of Multiple Prep and Reveal Networks, the proposed system was able to increase the security of our image retrieval model by incorporating noise-filled secret images into the original cover image rather than hiding them in LSBs of the original cover image. It has shown out to be an intriguing system to use many decoders to extract the decoded secret from a single encoded cover. This technique is an image-domain extension of the same concept. When employing this methodology, there is no requirement to reduce the size of an image or compromise the colour channels of the secret images. Although, contractual decoders are also possible to employ in place of multiple decoders, multiple prep networks and reveal networks were adopted for the implementation.Preparing and combining two secret images in chaotic image encryption serves to enhance security and complexity by introducing additional layers of information and complexity into the encryption process by making advantage of Enhanced Confusion and Diffusion, Redundancy and Error Tolerance and Algorithmic Complexity.

### Deep neural network model formulation

**Figure 3:**
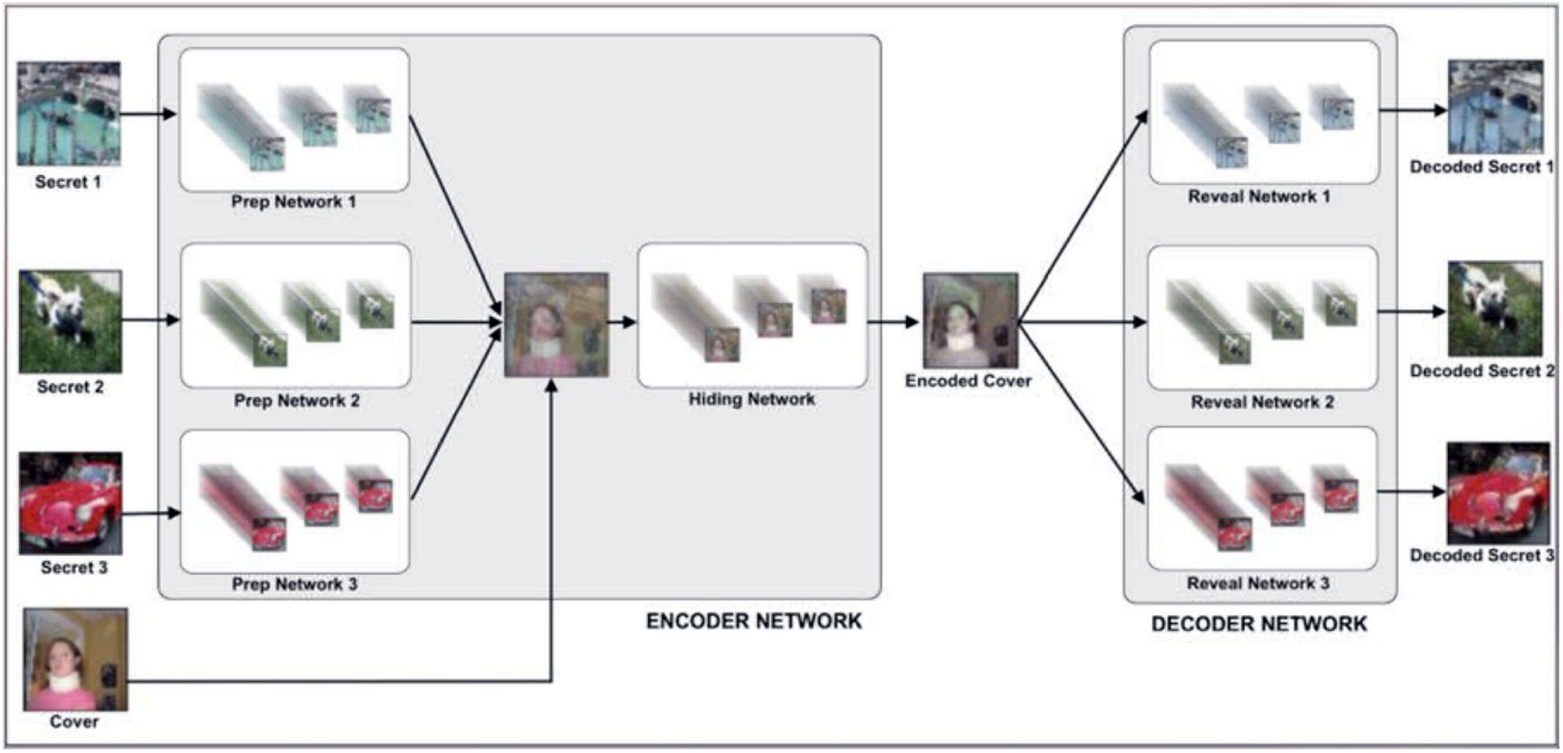
Deep neural network model formation.

The proposed system consists of major three layers: Layer 1: SRDNN for image super-resolution without feature loss; Layer 2: a steganographic technique for creating stegno images; Layer 3: a cryptographic technique for high security and the preparation networks is made up of two layers. Each layer of the preparation network is composed of three separate 2D Convolutional layers. The number of channels used in those three 2D Convolutional layers is 50, 10, and 5, respectively. Each layer employs kernel sizes 3, 4, and 5. The stride length was set to 1 along both axes. To keep the output image in the same dimensions, padding is added to each of the 2D Convolutional layers. Each 2D Convolutional layer is followed by an activation function which is most commonly used in neural networks called ReLU activation. Rectified Linear Unit is abbreviated as ReLU. It is a variational linear combination that will straightforwardly output the input if it is positive; otherwise, it will output zero. Figure 3 shows the Deep Neural Network model formation. The hiding network is composed of five layers. Each layer is composed of three separate 2D Convolutional layers. The fundamental structure of the Conv2D layers is similar to the Preparation Network’s Conv2D layers. The architecture of reveal networks is similar to that of the hiding network. It employs 5 layers of 2D Convolutional layers that are similarly formed.

### 3D chaotic mapping

In image encryption methods, 3D chaotic maps can be used to achieve confusion and diffusion. Confusion is the process of complicating the relationship between plaintext and ciphertext data, whereas diffusion is the process of spreading the influence of individual plaintext elements across the entire ciphertext. Without a valid decryption key, chaotic maps can be used to scramble the pixel positions of an image, making it difficult for unauthorised users to recognise the content. Image transmission through unsecured channels, such as the internet or wireless networks, can be secured using 3D chaotic maps. The encryption method ensures that the original image is only accessible to the intended recipient.

**Figure 4:**
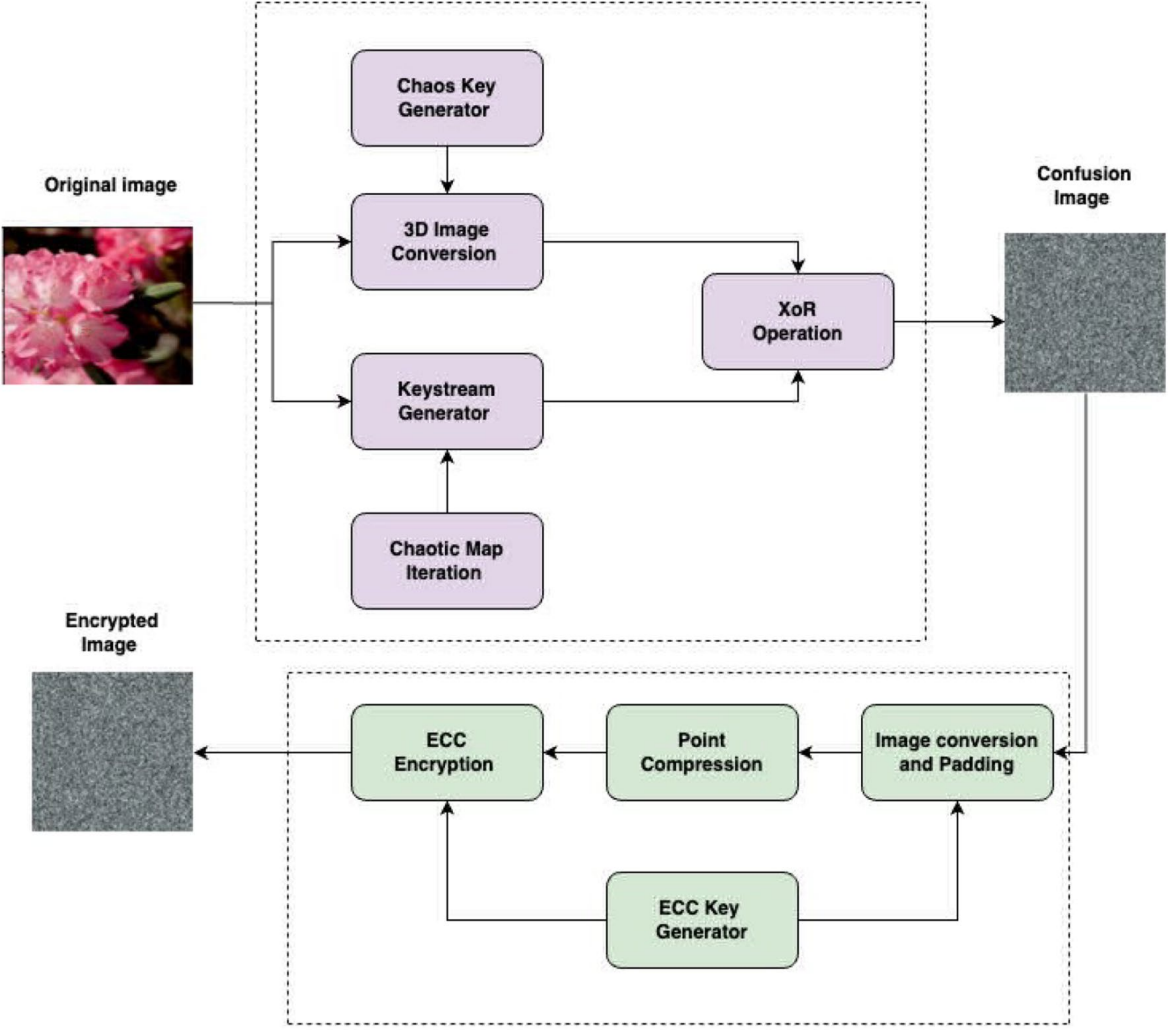
Chaotic and ECC process.

Figure 4 shows the steps involved in the chaotic and ECC process. The process is carried out by Embedding a chaos key into a 3D converted picture entails a procedure in which the chaotic sequence created by the key is utilized to change or transform the 3D image data. A 3D-chaotic system is used to produce a chaotic sequence. As a result, this sequence functions as the encryption key. Through a series of processes involving the 3D-chaotic sequence and the image matrices, the chaotic key is embedded in the 3D image data. The inbuilt chaotic key is utilized to execute 3D picture encryption and transformation operations. In the 3-D chaotic stage, during the Key Generation process, the initial conditions and parameters for the 3D chaotic map are generated. These parameters will be used as the encryption key. It’s crucial to keep this key secure as it will be required for decryption. In the Image Conversion process, the 3D medical image is converted into a suitable format for encryption. This might involve transforming the 3D image into a 1D or 2D array or dividing it into smaller blocks. In the Chaotic Map Iteration, the 3D chaotic map is iterated using the generated key to obtain a series of chaotic values. The 3D chaotic map will generate pseudo-random values based on the initial conditions and parameters. These values will form the keystream used for encryption. Later, a sequence of random numbers is extended from the chaotic map’s iterations during the Keystream Generation phase. These random numbers, also known as the keystream, will be used to XOR with the image data during encryption. A bitwise XOR is performed between the keystream and the image data in the XOR Encryption stage. This process is often performed on individual image pixels or blocks. The XOR operation effectively combines the randomness of the keystream with the image data, creating the encrypted image. These 3-D mapped images are then to ECC for cryptographic encryption. The proposed model incorporates a chaotic key generator that involves in creating a chaotic signal or sequence that serves as the encryption key. The chaotic system generates a pseudo-random sequence, often derived from a 3D-chaotic map. The key generation process is crucial as it forms the basis for encrypting and decrypting the image. After generating the chaotic key, the encryption process involves iterating this key with the image data. The chaotic key is combined with the image pixels using chaotic mathematical operations to scramble the image data. Iterating the chaotic key typically involves applying the chaotic sequence repeatedly to different parts or layers of the image, ensuring enhanced security.

### Elliptic curve cryptography

In the ECC process depicted in Fig. 4, a key is generated where a suitable elliptic curve and its parameters are chosen. The generated private key (a random number) is the secret key for encryption and the corresponding public key is based on the chosen elliptic curve and the private key is computed. Secondly, in the Image Conversion and Padding stage, the image is converted into a binary or numerical representation. This step is essential as ECC operates on numerical data. If necessary, padding is performed to ensure the image data matches the curve’s field size or encoding requirements. The Point compression stage is identified to be an optional stage in which the image points can be compressed using point compression techniques that reduce the size of the data to be an encrypted image^19^.

In the encryption stage, the image is divided into blocks if necessary for parallel processing. For each block, select a random ephemeral private key (nonce). The corresponding ephemeral public key for the selected nonce is computed. In an ECC point multiplication is performed between the ephemeral public key and the recipient’s public key. This results in a shared secret point on the elliptic curve. Then the shared secret point with the image block obtains the encrypted block through the XOR operation. Similarly in the decryption stage, The recipient possessing the private key corresponding to the public key used during encryption can decrypt the encrypted image blocks and the encryption process is repeated for each block to retrieve the original image data. To read the output of the image perfectly Image Reconstruction and Additional Security Measures can be applied so as to enhance security. This could be achieved by some ECC-based image encryption schemes being used as additional techniques, such as hybrid encryption (combining ECC with symmetric encryption algorithms), randomized padding, or message authentication codes (MACs) to detect tampering.

### Ethical approval

In complete adherence to established ethical research guidelines, it is imperative to emphasize that no experimentation involving either human subjects or animals took place during the specified study time as documented in this scholarly article. Our research primarily focused on the investigation of a metallic prosthesis, hence eliminating the need for any type of human trial. The methods employed in our study centered on non-invasive procedures and in vitro assays for data collection. The aforementioned methodology played a pivotal role in guaranteeing the ethical treatment of all entities capable of sentience, during study period.

## Experimental results and evaluations

The experimental analysis was carried out on the 11th Gen Intel® Core^TM^ i7-1165 G7@2.80Ghz with 8 GB RAM using MatlabR2023.To train and test the model, the Linnaeus 5 dataset is used. The dataset is divided into five classes, with a total of 6000 images for training. Of which 300 images are captured in order to train the model. Using the loaded dataset function, the images were extracted from the Linnaeus 5 dataset for further processing. This function takes 60 images from each class, a total of 300 images. The obtained 300 images are split into three sections namely: Secret Image 1, Secret Image 2, and Cover Image. Secret image 1 is captured in the first 100 images. The next 100 images are used to create secret image number two, and the surviving 100 images are used to create cover images. A hyperparameter controls how much the model changes in response to the approximated errors that occur when the model weights are modified. The Learning Rate is the name given to this hyperparameter. With, the learning rate remains steady for .0.01 till the first 200 epochs, 0.0003 from 200 - 400 epochs and 0.00003 for the remaining iterations respectively.

**Figure 5:**
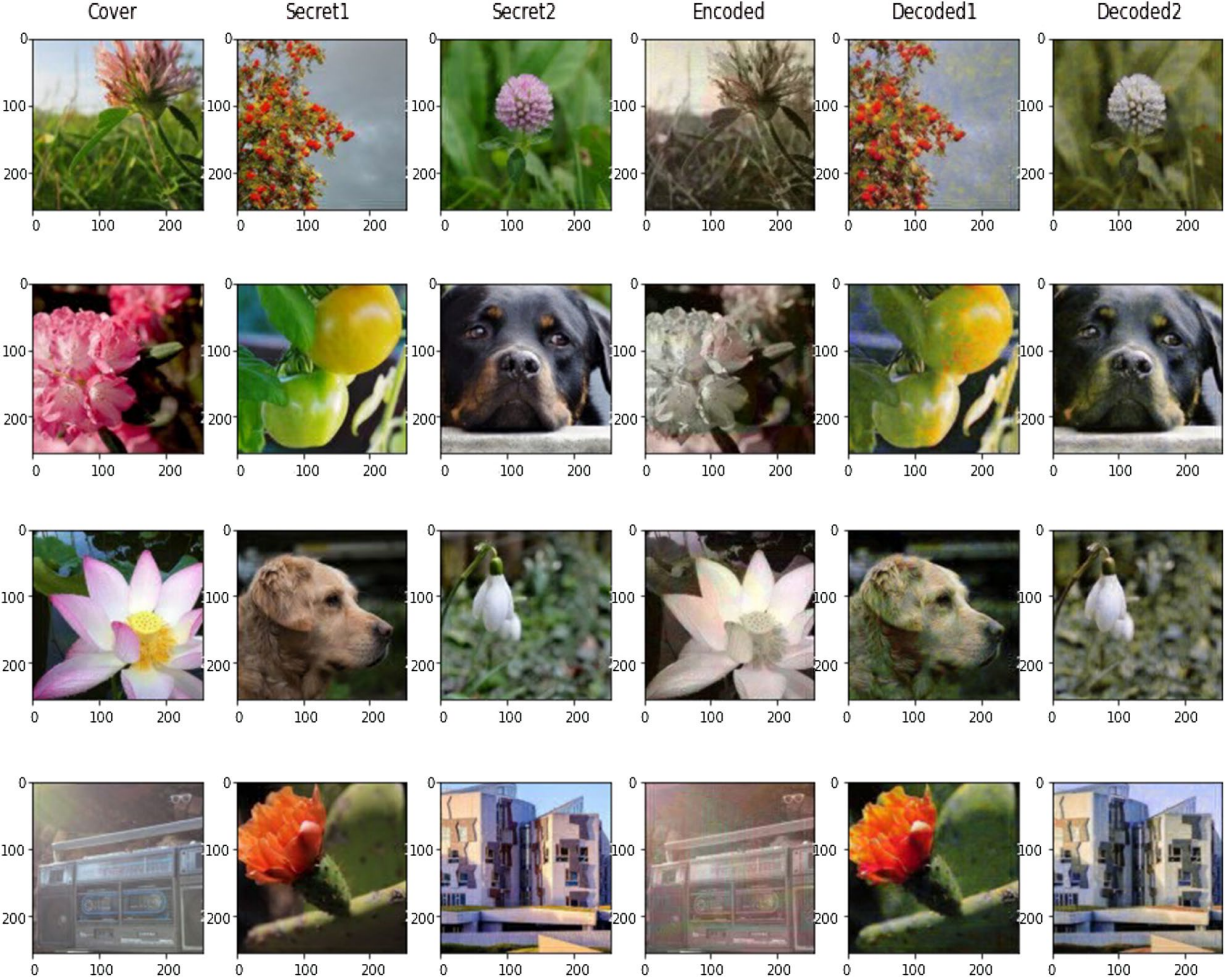
A sample of interpolated images.

The Model was trained over 300 epochs. The batch size in this case is ten. Image steganography techniques are evaluated using a variety of standardised measurements, including concealing capacity, recovery resemblance, concealing efficiency, and resistance to attacks. These assessments can be used to contrast the efficacy of different steganography techniques. Image metrics should fluctuate because many techniques vary markedly between methods and techniques, frameworks, and image datasets used. To reduce these fluctuations, the technique was applied to a wide range of images, from common to medical data images. Optimizers are algorithms or methods that change neural network attributes such as weights and learning rates to reduce losses. The Optimizer function is minimised to solve optimization problems.In this work, the Adam Optimization Technique is used. Adaptive Moment Estimation is the augmentation for ADAM thatis considered. Adam can be described as an amalgam of RMSprop and Stochastic Gradient Descent with momentum. Adam is used to approximating adaptive learning rates for each parameter. The experimental analysis of the proposed super-resolution approach to enhance the pixel density of low-quality input image data is considered, as are image quality assessment measures. The down-sampling method is used to generate low-resolution images during this training phase. The interpolated images are then up- sampled using the nearest neighbour with two skip connections and bilinear interpolation with upscaling factors ranging from 3 to 6. A sample of the interpolated images is shown in Fig. 5.

### Statistical analysis of the encrypted image

In order to ensure the high security of the encrypted image, various statistical tests are performed to investigate the strength of the proposed algorithm using chaotic mapping and ECC. The proposed framework is evaluated using Grayscale images from the acquired dataset. The analysis carried out includes the entropy of cipher images, measuring resistance to differential attacks with NPCR and UACI parameters, similarity measurements (PSNR), Structural similarity index (SSIM),normalized correlation coefficient (NC) and encryption and decryption time comparisons were all performed.

#### Encrypted image entropy

Information entropy is intrinsically linked to randomness measurement. According to Shannon’s entropy theory, entropy is directly proportional to the degree of uncertainty in the data. An encryption technique should ideally output a cipher image with an entropy value of 8 which is computed using the Eq. (1). In Table 1, the entropy values are computed for the cipher images derived from the proposed algorithm.

1 $$E\left( m \right) = - \sum\limits_{{i = 0}}^{{255}} {P\left( {m_{i} } \right)} \log _{2} \left( {P\left( {m_{i} } \right)} \right)$$

**Table 1:** Entropy computation of cipher images.

Test image	Entropy cipher
Image 1	7.9893
Image 2	7.9890
Image 3	7.9872
Image 4	7.9806

#### Differential attack analysis: NPCR and UACI

Differential attack analysis involves, crypt analysis to find the secret key by identifying the differences in the cipher image that result from slight adjustments to the plain image data. The resistance of the algorithm against such attacks is assessed using the Number of Changing Pixel Rates (NPCR) and Unified Average Changes Intensity (UACI) that are derived from the cipher image obtained from a pair of near identical plain images through Eqs. (2), (3) and (4) respectively. The resistance to attack is ideally accepted when the NPCR value for encryption is close to 100%, whereas the UACI value must be closely mapped to 34% which is found to be the same in the proposed methodology. The NPCR and UACI values of the proposed encrypted mechanism are tabulated in Table 2.The author has presented a method for transferring grayscale images that is extremely secure, with near-zero correlation and an Entropy closer to 8. The Peak Signal Noise Ratio (PSNR) for a directly decrypted picture with an embedding capacity of 0.0807 bpp was measured to be 50.84 dB. Furthermore, the secret and cover photos are correctly recovered^20^.

2 $$\begin{aligned} \text {NPCR}= & {} \frac{\sum _{i=1}^{m} \sum _{j=1}^{n} f(i,j)}{m \cdot n} \times 100\% \end{aligned}$$

3 $$\begin{aligned} \text {UACI}= & {} \frac{\sum _{i=1}^{m} \sum _{j=1}^{n} \left| I'(i,j) - I''(i,j) \right| }{255 \cdot m \cdot n} \end{aligned}$$

4 $$\begin{aligned} f(i,j)= & {} \bigg \{ \begin{array}{ll} 0 &{} \text {if } E(i,j) = E'(i,j) \\ 1 &{} \text {if } E(i,j) \ne E'(i,j) \end{array} \end{aligned}$$

**Table 2:** Computations of differential attack analysis.

Test image	NPCR	UACI
Image 1	99.5428	33.4456
Image 2	99.5412	33.4442
Image 3	99.5401	33.4426
Image 4	99.5426	33.4438

#### Similarity measurement: PSNR analysis

The similarity between the plain and the cipher images is determined by the Peak- Signal- to- Noise Ratio (PSNR) and structural similarity index values using Eq. (5). The PSNR decreases as the mean squared error (MSE) gets closer to 0. A significant distinction between the original and encrypted image seen by looking at the lower PSNR values. Table 3 displays the PSNR values for different grey and color images.

5 $$\begin{aligned} PSNR=20\times log_{10} \frac{P_{max}}{\sqrt{MSE}} \end{aligned}$$

**Table 3:** Computation of PSNR value.

Test image	PSNR value
Image 1	5.4432
Image 2	6.2834
Image 3	6.7846
Image 4	6.3782

### NIST-SP 800-22 analysis

The NIST test is run on the cipher image to verify the encryption randomness. The proposed method analysis the resulting cipher image randomness using NIST test which consist of 15 distinct tests. If the p value is more than 0.01 for a cipher image, it is considered to be random. The experimental results are observed as in Table 4.

**Table 4:** Computations of differential attack analysis.

Test name	P-value	Result
Frequency	0.528079	PASS
Block frequency	0.801252	PASS
Cumulative sums	0.411586	PASS
Runs	0.501297	PASS
Longest run of ones	0.180611	PASS
Rank	0.741968	PASS
DFT	0.027896	PASS
Non-periodic template matches	0.341454	PASS
Approximate entropy	0.221968	PASS
Random excursions	0.500259	PASS
Random excursion variant	0.681901	PASS
Serial	0.591654	PASS
Linear complexity	0.854508	PASS
Overlapping template matchings	0.2648	PASS
Universal statistical	0.7678	PASS

### Robustness analysis of noise and cropping attacks

The robustness of the proposed image encryption technique is evaluated based on its ability to resist noise and the recipient’s capacity to identify the image once it has been recovered. The proposed scheme is tested by adding 0.01 and 0.005 density salt and pepper noise and gaussian noise to the encrypted image in Fig. 4. So based on the Table 5 results, it is evident that the suggested scheme is extremely resistant the noise attack. In order to test the robustness of the cropping attack, the encrypted image is tested with the crop size of 64 × 64, 128 × 128 and 256 × 256. Despite the cropping attack the image data is still recognizable and it exhibits the good cropping robustness of the proposed technique as in Table 6.

Tables 5 and 6 examines the PSNR, SSIM and NC values for encrypted image with cropping , salt and pepper noise and gaussioan noise. The PSNR, SSIM and NC values in Tables 5 and 6 are almost greater than 59 decibel and close to 1 respectively, indicating that the proposed method is capable of resisting both cropping and noise attack.

**Table 5:** Noise attack analysis.

Attack	Noise density	PSNR	SSIM	NC
Salt and pepper noise	0.005	60.58	0.9956	1
	0.01	60.71	0.9995	0.9986
Gaussian noise	0.0001	61.32	0.9994	1
	0.0005	59.98	1	0.9989

**Table 6:** Cropping attack analysis.

Cropping attack			
Crop size	PSNR	SSIM	NC
64x64	59.88	0.8858	0.9988
128x128	59.25	0.8974	0.9687
256x256	59.59	0.9954	0.9929

#### Time complexity analysis

The encryption and decryption analysis were performed using a modified ECC algorithm. The primary time-consuming operation for the ECC-based image encryption technique is point multiplication. The time complexity of the proposed methodology is found to be minimal because of the reduction made in the point-multiplication operations. The execution time of the encryption and decryption is tabulated as in Table 7.

**Table 7:** Computation of Time Complexity.

Test image	Encryption (in sec)	Decryption (in sec)
Image 1	1.44608	1.10132
Image 2	1.43368	1.12146
Image 3	1.42646	1.11018
Image 4	1.43791	1.11007

#### Loss analysis

The loss function in the neural network is used to quantify the variance between the anticipated outcome and the outcome generated by the machine learning model^13^. The weights are updated using the gradients. The gradients can be calculated using the loss function. The overall average losses are utilized to determine the cost. The Mean Sum of Squared Error is used to calculate the loss. The two losses calculated are the final model loss and reveal network loss. The loss of the entire model, including that of the loss in the cover image and the loss procured in the secret image, is wrapped by full model loss and tabulated in Table 8. The loss accumulated only for secret information retrieval is referred to as reveal network loss. The loss used for the full model is given by Eq. (6)

6 $$\begin{aligned} \lambda = \lambda _c \cdot \left\| C - C'\right\| ^2 + \lambda _{s1} \cdot \left\| S1 - S1'\right\| ^2+ \lambda _{s2} \cdot \left\| S2 - S2'\right\| \end{aligned}$$

where $\lambda _c, \lambda _{s 1}, \lambda _{s 2}$ are the Beta values which is taken as 1.0: $\text{C}$ original cover image, C′ predicted cover image, $\text{S} 1$ original secret image 1, S2 original secret image 2, S1′ predicted secret image 1, S2′ predicted secret image 2.

**Table 8:** Computation of model Loss.

Total loss	46649.1
Loss in secret Image 1	8791.78
Loss in secret Image 2	8441.73

The model’s loss is initially extremely significant, but after training, the results are preferable to the original versions.

**Figure 6:**
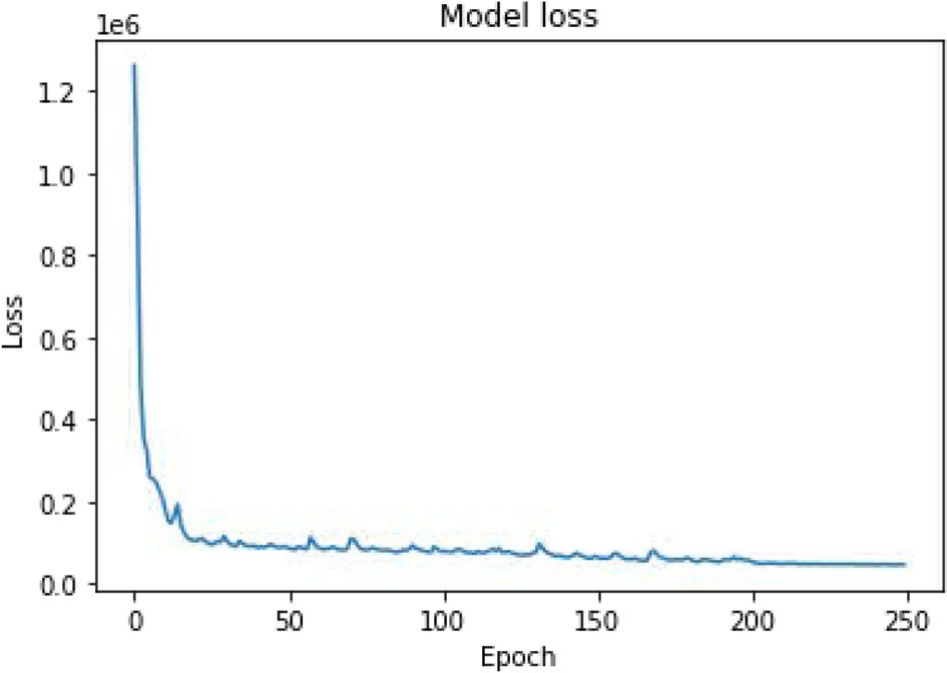
Loss curve of proposed model.

Figure 6 depicts that the loss of the proposed model is decreased as the number of epochs increases. The loss is also identified to vary on a minimal scale when the number of epochs is greater than 50 which indicates that the model is free from overfitting and underfitting. The hyperparameters are tuned in a way that the loss is minimal as the model is trained with larger feature inputs.

## Conclusion

The secret images obtained from the cover image resemble the original image in appearance. Because the model was trained with fewer iterations, the pixel loss is slightly higher. After examining the outcomes, it is noticed that far more loss occurs in images with greater red values while it is lower in green values of images. The results are going to be substantially better if the model is trained with a larger number of iterations than we have obtained. The model can be well trained to recover the secret image as from cover image by determining an optimal learning rate.When the images are encrypted before hiding into the cover image using 3D chaotic mapping and ECC, it is impossible to decrypt it back to the original image. It is because the neural network involves losses and some data of the encrypted content are lost because of the loss of the neural network. So, the padding of the content gets altered and it is impossible to decrypt using 3D chaotic mapping and ECC.The primarily focus is on the visual perception of images and not on the model testing with other types of losses. If the model’s loss is reduced by finding an optimal loss function and selecting the optimal learning rate, and the model is well-trained by boosting the total amount of iterations, this will end up serving as one of the appropriate response to hide images using steganography when compared to other conventional methods. Also, the encryption layer can be used before the preparation network, increasing the security of secret images by using other cryptographic algorithms that work even when there is a loss of secret images. On a general context, this paper is an effort to show that the principles of cryptography and steganography when effectively utilized, go hand in hand in conserving the privacy of the data.

## Data Availability

A sample space of the data considered for simulation is outlined in Table 4.
